# Synthesis and Characterization of CuO Nanoparticles with Differential Antifungal Activity Against *Fusarium graminearum* and *Trichoderma harzianum*

**DOI:** 10.3390/nano16211396

**Published:** 2026-10-31

**Authors:** Brenda Arriaga-García, Reyna Isabel Rojas-Martínez, Ana María Hernández-Anguiano, Víctor Manuel Zúñiga-Mayo, Julio Huerta Espino

**Affiliations:** 1 Posgrado en Fitosanidad-Fitopatología, Colegio de Postgraduados, Carretera México-Texcoco Km 36.5, Montecillo, Texcoco C.P. 56264, Estado de México, Mexico; arriaga.brenda@colpos.mx (B.A.-G.); ahernandez@colpos.mx (A.M.H.-A.); 2 Secretaría de Ciencia, Humanidades, Tecnología e Innovación (SECIHTI)-Posgrado en Fitosanidad-Fitopatología, Colegio de Postgraduados, Carretera México-Texcoco Km 36.5, Montecillo, Texcoco C.P. 56264, Estado de México, Mexico; 3 Programa de Trigo, Campo Experimental Valle de México, Instituto Nacional de Investigaciones Forestales, Agrícolas y Pecuarias (INIFAP), Carretera los Reyes-Texcoco km 13.5, Texcoco C.P. 56250, Estado de México, Mexico; espino.julio@outlook.com

**Keywords:** antifungal activity, biological control, copper oxide nanoparticles, *Fusarium graminearum*, *Trichoderma harzianum*

## Abstract

Copper oxide nanoparticles (CuO NPs) have attracted increasing interest in agriculture due to their antimicrobial properties and potential for sustainable plant disease management. In this study, CuO NPs were synthesized via chemical precipitation, and their antifungal activity and morphological impacts were evaluated against the phytopathogen *Fusarium graminearum* and the biocontrol agent *Trichoderma harzianum*. Comprehensive physicochemical characterization confirmed the formation of highly pure, quasi-spherical CuO NPs with an average size of 10.9 ± 0.7 nm as determined by TEM. Furthermore, Raman spectroscopy and X-ray diffraction (XRD) revealed a highly crystalline monoclinic tenorite phase (space group C2/c) with an average crystallite size of 9.6 ± 0.7 nm, confirming the single-crystal nature of the nanomaterial. The CuO NPs exhibited marked antifungal activity against *F. graminearum*, achieving 100% growth inhibition at 500 ppm, accompanied by severe hyphal deformation and cell wall collapse. In contrast, *T. harzianum* showed tolerance, maintaining growth up to 800 ppm. SEM analysis revealed that under sublethal stress, both fungi differentiated vegetative mycelium into chlamydospores as a survival strategy. Furthermore, a low concentration (50 ppm) of CuO NPs stimulated *T. harzianum* sporulation by 30.4% relative to the control, consistent with a hormetic-type response. These findings highlight a differential sensitivity of the two fungi to CuO NPs *in vitro*. While the nanoparticles effectively inhibit the phytopathogen, the beneficial biocontrol fungus exhibits remarkable tolerance. Although these results provide a promising basis for exploring the combined use of CuO NPs and *T. harzianum*-based biofungicides, further in vivo studies evaluating this interaction with the plant are necessary to confirm their compatibility and efficacy in sustainable integrated disease management.

## 1. Introduction

Nanotechnology (NT) involves the design and modification of materials with dimensions ranging from 1 to 100 nm, which may exhibit physical, chemical, and biological properties distinct from those of their bulk counterparts, thereby expanding their applications across various fields [1,2,3,4,5]. In agriculture, nanomaterials (NMs) have emerged as strategic tools for improving the productivity, sustainability, and resilience of agricultural systems through applications such as nanobiosensors, nanofertilizers, bioremediation, and crop protection [6,7,8,9].

*Fusarium graminearum* is the primary causative agent of *Fusarium* head blight (FHB) in wheat (*Triticum aestivum* L.), a globally important disease that reduces yield and grain quality [10]. Furthermore, infection promotes the accumulation of mycotoxins, including trichothecenes such as nivalenol and deoxynivalenol, thereby compromising food safety [11,12]. Since no commercial cultivars exhibit complete resistance to FHB, the development of complementary strategies for controlling *F. graminearum* remains necessary [11,13].

The intensive use of conventional agrochemicals has led to problems associated with environmental contamination, reduced efficacy, and the development of resistance in plant pathogens [14,15,16]. In this context, metal oxide nanoparticles (NPs) have attracted considerable interest due to their antimicrobial properties, which are primarily associated with the generation of reactive oxygen species (ROS), the release of metal ions, and the disruption of cellular structures [17,18,19]. In particular, CuO NPs have demonstrated inhibitory activity against various phytopathogenic fungi, including species of *Penicillium*, *Alternaria*, *Aspergillus*, and *Fusarium*, due to their ability to induce oxidative stress, membrane damage, and intracellular alterations [20,21,22,23,24]. However, the impact of antimicrobial nanomaterials on beneficial microorganisms used in agriculture remains insufficiently studied.

On the other hand, species of the genus *Trichoderma*, particularly *T. harzianum*, are widely used as biological control agents due to their diverse mechanisms of action, including mycoparasitism, competition for nutrients, and induction of plant resistance [25,26,27]. Their high adaptability, ability to produce spores, and efficacy under different agricultural conditions have facilitated their development as commercial biofungicides [28,29,30,31]. Furthermore, advances in fungal genetic engineering technologies offer new opportunities for developing improved strains with enhanced biocontrol activity, contributing to the advancement of more sustainable agricultural practices [32].

Fungi used as biological control agents must possess specific biological traits that ensure their effectiveness and viability. Among these, reproducible antagonistic activity, genetic stability, environmental and human safety, and the capacity for large-scale production of viable conidia are particularly important for the development of commercial formulations [33,34,35,36]. Consequently, evaluating the compatibility of antimicrobial nanomaterials with beneficial fungi is essential when considering their potential integration into biological control strategies.

A previous study has demonstrated the inhibitory effects of CuO nanoparticles at 100 ppm on *F. graminearum*, including alterations in hyphal growth, conidial viability and germination, oxidative stress, and cell membrane integrity [37]. In addition, Banik and Pérez-de-Luque reported that metallic copper nanoparticles at 100 ppm did not significantly inhibit the mycelial growth of *T. harzianum,* whereas the same concentration accelerated and increased its sporulation rate under their experimental conditions [38]. It has also been reported that CuO NPs at concentrations of up to 200 ppm did not affect the growth of *T. asperellum*; however, a delay in conidiation was observed at 100 and 200 ppm [39]. These findings provide important insights into the effects of copper-based nanomaterials on pathogenic and beneficial fungi; however, the response may differ depending on the specific composition of the nanoparticle and the fungal species analyzed. Furthermore, although individual studies exist, there is a lack of direct, comparative evaluations under identical conditions to understand the differential sensitivity between a major phytopathogen and a beneficial biocontrol agent.

Therefore, this study aimed to synthesize and characterize CuO nanoparticles and comparatively evaluate their concentration-dependent effects on *F. graminearum* and *T. harzianum* under the same *in vitro* experimental conditions. By assessing fungal growth, morphological changes, and sporulation, this study investigates the differential sensitivity of a wheat pathogen and a beneficial biocontrol fungus to the same characterized nanomaterial. This comparative approach provides a basis for discussing the potential use of CuO nanoparticles in agricultural disease management while considering their effects on beneficial microorganisms.

## 2. Materials and Methods

### 2.1. Synthesis of CuO NPs by Chemical Precipitation

CuO NPs were synthesized using the chemical precipitation method previously described by Lanje et al. [40], with modifications. Briefly, 300 mL of a 0.02 M copper acetate solution (MEYER^®^, Mexico City, Mexico) was maintained under constant stirring, and 1 mL of glacial acetic acid (MEYER^®^, Mexico City, Mexico) was added to control precipitation. The solution was heated to 100 °C, after which 5 M NaOH (Sigma-Aldrich, St. Louis, MO, USA) was added until a pH of 6.5–6.7 was reached. The suspension was allowed to stand for 24 h at room temperature to promote nanoparticle precipitation.

The resulting precipitate was recovered by centrifugation at 3500 rpm (Velospin 22R, Cryste, Seoul, South Korea) for 30 min. Subsequently, the precipitate was washed twice with distilled water, followed by a wash with absolute ethanol, with the supernatant discarded after each centrifugation. Finally, an additional wash with distilled water was performed. In all cases, the samples were centrifuged at 3500 rpm for 20 min. The recovered material was dried at room temperature, weighed, and stored until characterization. The synthesis was performed independently in triplicate. The theoretical yield of CuO was calculated from the stoichiometric relationship between Cu(CH_3_COO)_2_ and CuO and their respective molar masses. The synthesis yield was calculated as the ratio of the experimentally recovered CuO mass to the theoretical CuO mass, multiplied by 100 [41].

### 2.2. Physicochemical Characterization of CuO NPs

The morphology, size, and elemental composition of the CuO NPs were analyzed using scanning electron microscopy (SEM; JEOL JSM-6390, JEOL Ltd., Tokyo, Japan) and transmission electron microscopy (TEM; Tecnai G2 Spirit TWIN, FEI Company, Hillsboro, OR, USA) operated at an accelerating voltage of 120 kV. Prior to analysis, the nanoparticles were dispersed by sonication (Branson 1800, Branson Ultrasonics Corp., Danbury, CT, USA) for 30 min in acetone (J.T. Baker, Avantor, Inc., Radnor, PA, USA). For TEM analysis, the suspension was deposited onto copper grids coated with Formvar–carbon (200 mesh, Agar Scientific Ltd., Stansted, UK) and allowed to dry at room temperature.

The average primary particle size was determined by analyzing approximately 600 nanoparticles using ImageJ v1.54d [42]. The size distribution was fitted to a log-normal function using OriginPro 8.5.0 SR1 2022 [43]. Elemental analysis was performed using energy dispersive spectroscopy (EDS).

The crystalline structure was evaluated using Raman spectroscopy and X-ray diffraction (XRD). Raman spectra were obtained using a 633 nm excitation wavelength and compared with the RRUFF database using the tenorite phase (R120076) as a reference. Analysis of the Raman bands was performed using Fityk 1.3.1 software [44]. XRD patterns were recorded over a range of 20–80° and analyzed by Rietveld refinement using FullProf Suite (version July 2017), considering the monoclinic structure of CuO (space group C2/c) [45,46]. Finally, microstructural effects were evaluated using the spherical harmonics method, the Thompson–Cox–Hastings pseudo-Voigt function, and the Debye–Scherrer equation [47].

### 2.3. Preparation of Culture Media with NPs

Potato dextrose agar (PDA; Difco^TM^ Becton, Dickinson and Company, Franklin Lakes, NJ, USA) was prepared according to the manufacturer’s instructions and sterilized by autoclaving. To prepare the treatments, the required mass of copper oxide nanoparticles (CuO NPs) was suspended in 0.1 L of sterile distilled water and dispersed via sonication for 20 min without adding surfactant or stabilizing agents. Following PDA autoclaving, the NP suspension was incorporated into the molten PDA and adjusted to a final volume of 1 L. Distinct concentration ranges were selected based on the biological response evaluated: nominal concentrations of 50–800 mg L^−1^ (50, 200, 300, 400, 450, 500, 600, 700, and 800 mg L^−1^; equivalent to ppm) were chosen for mycelial growth assays to assess fungal response across a broad spectrum, whereas a lower range of 25–200 mg L^−1^ (in 25 mg L^−1^ increments) was applied in sporulation assays to analyze conidial production at lower exposures. To maintain homogeneous dispersion, the mixture was stirred continuously on a hot plate at 70 °C for 15 min prior to pouring into Petri dishes. In parallel, PDA Petri dishes without CuO NPs were prepared and used as controls. Nanoparticle aggregation and dispersion stability were not monitored during incubation.

### 2.4. Evaluation of Antifungal Activity and Morphological Analysis by SEM

To evaluate antifungal activity, 6-mm-diameter mycelial discs from 6-day-old cultures of *T. harzianum* and *F. graminearum* were placed on PDA supplemented with different concentrations of CuO NPs. Each treatment consisted of three independent Petri dishes per fungal species, with each plate serving as an experimental unit, and the experiment was repeated independently on two occasions. The dishes were incubated at 28 °C under a 12-h photoperiod. Radial growth was evaluated after 5 days of incubation, when the untreated control had completely covered the Petri dish, and plates were maintained under the same experimental conditions for a total period of 7 days. Radial growth measurements from each plate were used to calculate the percentage of mycelial growth inhibition according to González et al. [48].

To evaluate hyphal morphological changes, scanning electron microscopy (SEM; JEOL JSM-6390) was performed. Hyphal material was harvested from four Petri dishes per treatment by dividing each dish into four quadrants and collecting three random subsamples per quadrant to yield composite biological samples. Four independent SEM sample preparations were processed per treatment. From the resulting micrographs, the width of 400 hyphae per treatment was measured using ImageJ v1.54d software. All measurements were registered alongside their corresponding sample-preparation identifiers to preserve the hierarchical sampling structure and clearly differentiate microscopic subsamples from independent experimental replicates. Sample preparation was performed following the protocol described by Minuti et al. [49], with some modifications. The samples were fixed with 3% glutaraldehyde in Sorensen’s phosphate buffer (0.1 M, pH 7.2) (J.T. Baker, Avantor, Inc., Radnor, PA, USA), washed twice with the same buffer, and post-fixed with 1% osmium tetroxide (Sigma-Aldrich, St. Louis, MO, USA) for 24 h. Subsequently, the samples were washed twice with distilled water and centrifuged for 3 min at 12,000 rpm. Dehydration was carried out gradually with ethanol (J.T. Baker, Avantor, Inc., Radnor, PA, USA) (30–100%), in 10% increments every 20 min, including two final steps with 100% ethanol. Finally, the samples were dried to the critical point (Samdri-780A, Tousimis, Rockville, MD, USA), mounted on holders, and coated with a gold–palladium alloy (JFC-1100, JEOL Ltd., Tokyo, Japan)for observation by SEM.

### 2.5. Evaluation of Trichoderma harzianum Sporulation

The sporulation capacity of *T. harzianum* in the presence of CuO NPs was evaluated using 5-mm-diameter mycelial discs placed in Petri dishes containing PDA supplemented with different concentrations of CuO NPs (25–200 ppm). The Petri dishes were incubated at 28 °C in the dark for 10 days, with ten replicates per concentration. At the end of the incubation period, the conidia produced were harvested and quantified using a Neubauer chamber [50]. Subsequently, the conidial suspensions were adjusted to a concentration of 1 × 10^6^ conidia·mL^−1^ and inoculated onto PDA. The Petri dishes were incubated at 28 °C for 72 h to assess the germination capacity of the conidia [51].

## 3. Results

### 3.1. Synthesis of CuO Nanoparticles by Chemical Precipitation

CuO NPs were synthesized by chemical precipitation from copper(II) acetate, according to the reactions shown in Equations (1) and (2). Three independent syntheses were carried out at pH 6.6. In each synthesis, 1.198 g of copper(II) acetate was used, producing an average of 0.45 g of CuO NPs and a yield of 94.3%. The resulting samples were characterized using SEM, TEM, Raman spectroscopy, and XRD.

(1) $$Cu{\left(CH_{3}COO\right)}_{2}+2NaOH\to Cu{\left(OH\right)}_{2}↓+2CH_{3}COONa$$

(2) $$Cu{\left(OH\right)}_{2}\;\overset{∆}{\to }CuO+H_{2}O$$

Initially, copper(II) acetate reacts with NaOH to form insoluble copper(II) hydroxide (Equation (1)). Subsequently, through a process of thermal dehydration, the copper(II) hydroxide is converted to copper oxide (CuO) (Equation (2)).

### 3.2. Morphological Characterization by SEM and TEM and Elemental Analysis by EDS

**Figure 1:**
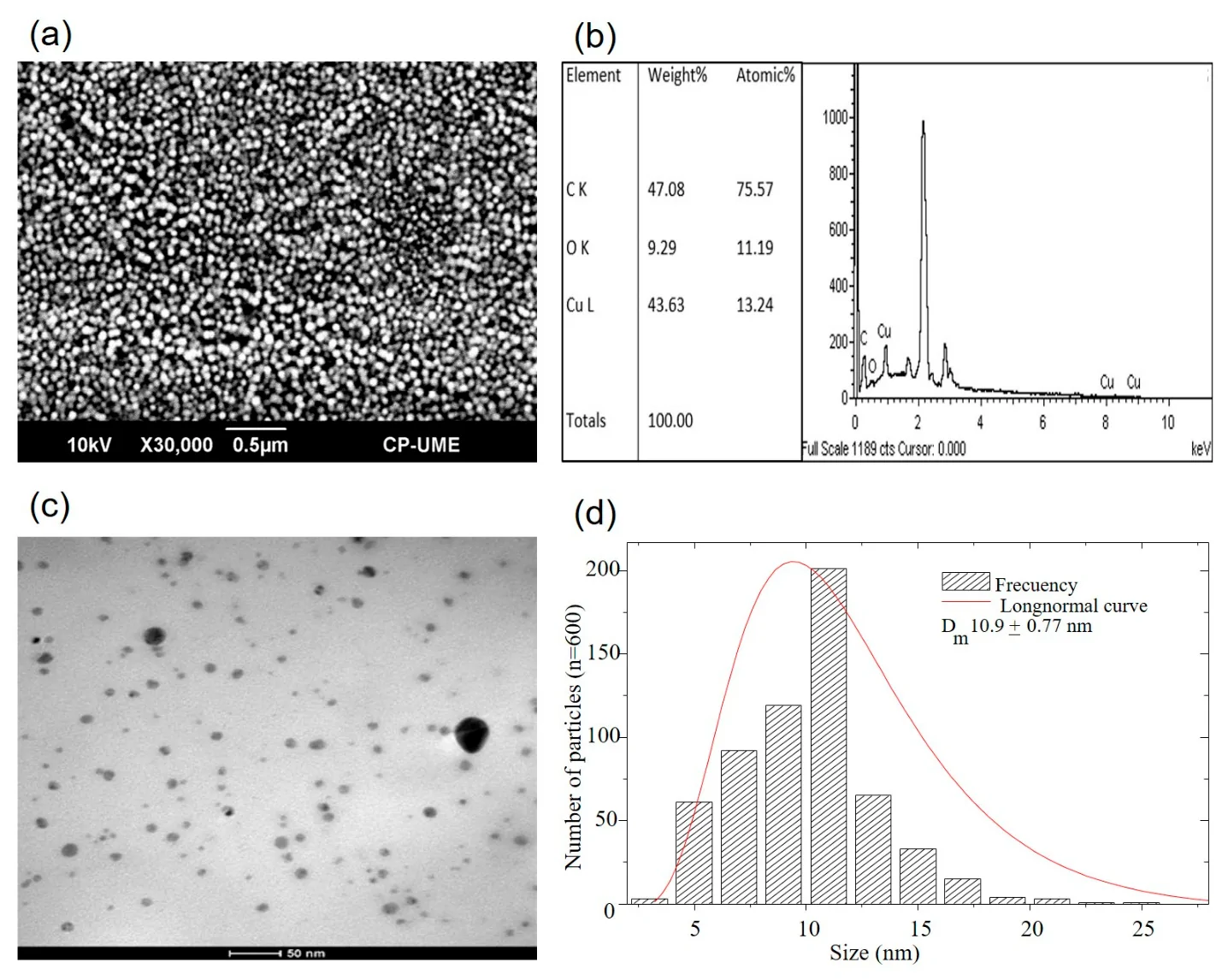
Morphological characteristics and elemental composition of CuO NPs synthesized by chemical precipitation. (**a**) Scanning electron microscopy (SEM-JEOL JSM-6390) micrograph of quasi-spherical CuO NPs acquired at 30,000× magnification; (**b**) elemental composition of the CuO NPs determined by energy dispersive spectroscopy (EDS), showing copper (Cu) and oxygen (O); (**c**) bright-field transmission electron microscopy (TEM-FEI Tecnai G2 Spirit TWIN 120 kV) micrograph of CuO NPs; and (**d**) particle size frequency histogram. The histogram was fitted using a log-normal distribution with a standard deviation of 0.77 nm.

The SEM micrographs showed that the CuO NPs exhibited a predominantly quasi-spherical morphology (Figure 1a and Figure S1). Elemental analysis by EDS confirmed the presence of Cu and O, indicating the high purity of the synthesized material. The presence of carbon was likely associated with the carbonaceous substrate used to adhere the nanoparticles (Figure 1b).

The TEM images corroborated the morphology observed by SEM (Figure 1c). Analysis of approximately 600 nanoparticles revealed an average particle size of 10.9 ± 0.7 nm, with a size distribution ranging from 2 to 25 nm (Figure 1d).

### 3.3. Characterization by Raman Spectroscopy and X-Ray Diffraction (XRD)

**Figure 2:**
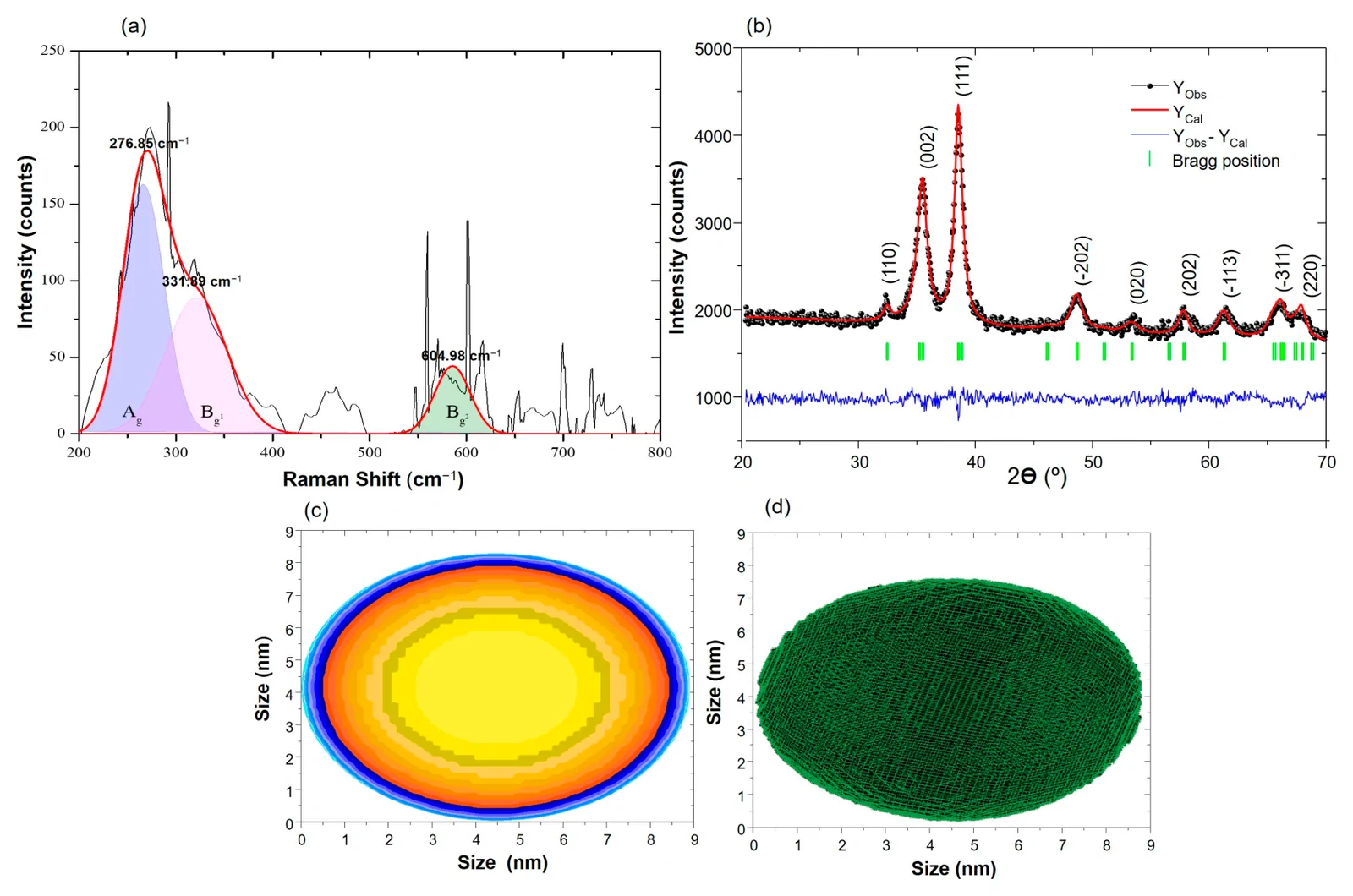
Structural characterization of CuO NPs synthesized by chemical precipitation using Raman spectroscopy and X-ray diffraction (XRD). (**a**) Raman spectrum of the CuO NPs and assignment of the characteristic vibrational modes; (**b**) XRD pattern of the CuO NPs with Rietveld refinement performed using FullProf software; and size and morphology models of the CuO NPs obtained using the spherical harmonics method with a (**c**) two-dimensional (2D) model and (**d**) three-dimensional (3D) model.

The Raman spectra showed three bands characteristic of monoclinic CuO, corresponding to the A_g_ (276.8 cm^−1^), B_g1_ (331.9 cm^−1^), and B_g2_ (605.0 cm^−1^) vibrational modes, confirming the formation of the tenorite phase (Figure 2a).

Rietveld refinement of the XRD patterns confirmed a monoclinic structure (space group C2/c), with lattice parameters of *a* = 4.6975 Å, *b* = 3.4143 Å, and *c* = 5.1537 Å, and a preferential orientation along the (111) plane (Figure 2b). The average crystallite size was 9.6 ± 0.7 nm, as calculated using the Debye–Scherrer equation. Spherical harmonic analysis yielded two-dimensional and three-dimensional models consistent with an ellipsoidal morphology (Figure 2c,d).

### 3.4. Effect of CuO Nanoparticles on Fusarium graminearum and Trichoderma harzianum

**Figure 3:**
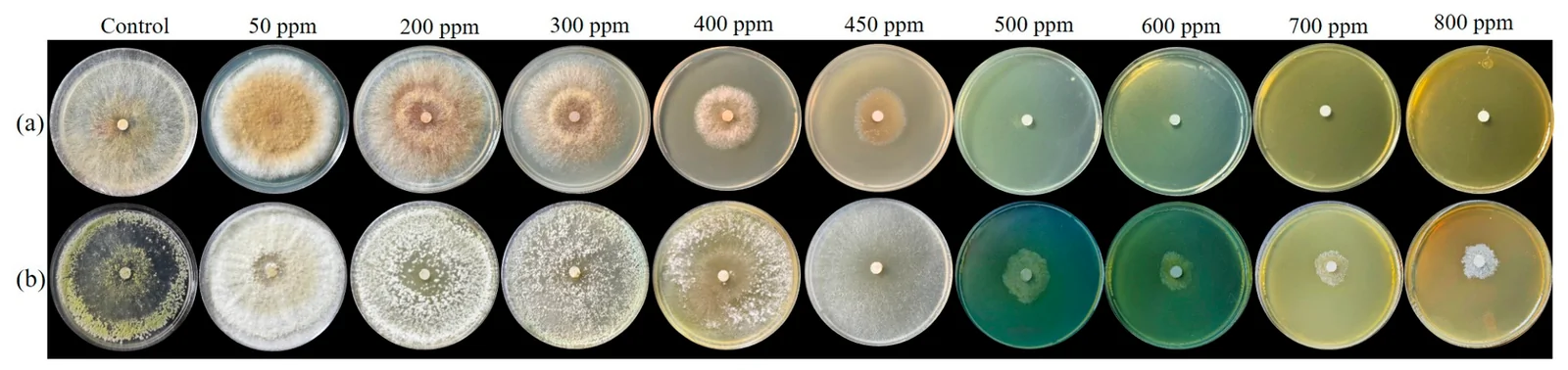
Mycelial growth of *F. graminearum* (**a**) and *T. harzianum* (**b**) on Difco™ potato dextrose agar (PDA) medium after 5 days of incubation in the presence of different concentrations of CuO NPs.

**Figure 4:**
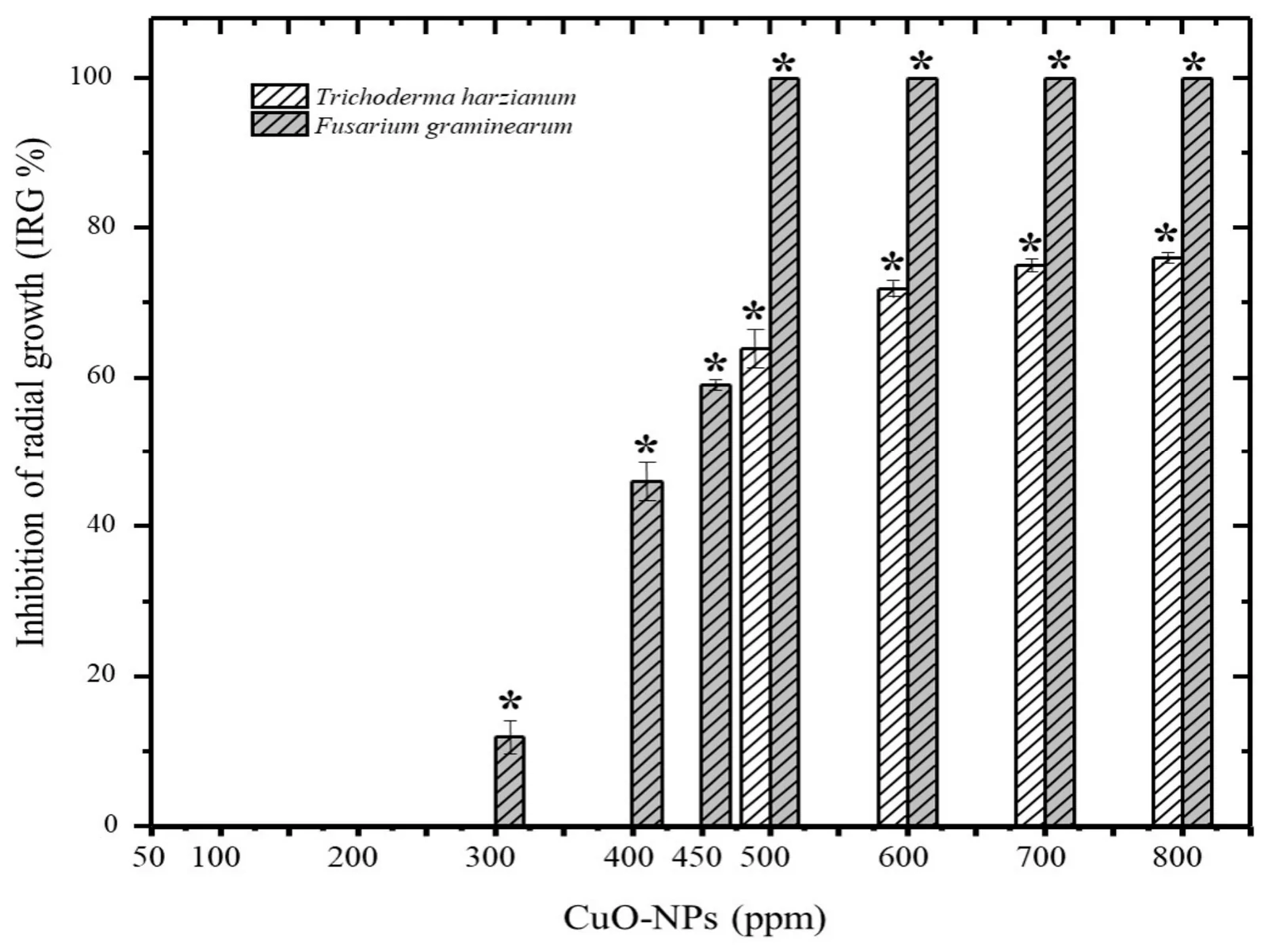
Radial growth inhibition (RGI, %) of *F. graminearum* and *T. harzianum* after 5 days of incubation in the presence of different concentrations of CuO NPs. * Indicates significant differences from the respective controls (without CuO NPs) according to Tukey’s test (*p* < 0.05).

The antifungal activity of CuO NPs was evaluated based on the inhibition of radial growth of *F. graminearum* and *T. harzianum* (Figure 3 and Figure 4). In *F. graminearum*, inhibition was first observed at 300 ppm (12%) and increased in a concentration-dependent manner, reaching 59% inhibition at 450 ppm and 100% at 500 ppm.

In contrast, *T. harzianum* showed greater tolerance to CuO NPs. Growth inhibition became evident at 500 ppm (64%); however, the fungus maintained mycelial growth even at 800 ppm.

**Figure 5:**
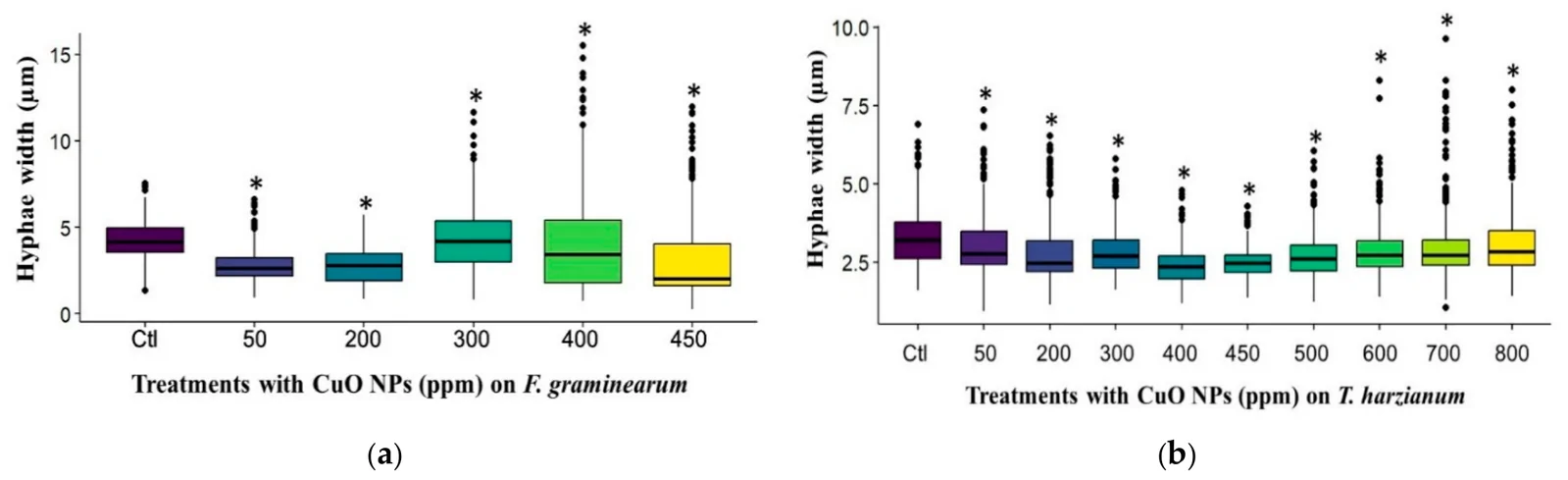
Hyphal width of fungi exposed to different concentrations of CuO NPs. (**a**) Mean hyphal width of *F. graminearum* and (**b**) mean hyphal width of *T. harzianum*. * Indicates significant differences from the respective controls according to the Kruskal–Wallis test (*p* < 0.05; *n* = 400).

To further characterize the antifungal effect of CuO NPs on both fungi, a phenotypic analysis of the hyphae was performed using SEM. Analysis of hyphal width showed significant differences between the treatments and the control for both species (Figure 5). In *F. graminearum*, high variability in hyphal width was observed; concentrations of 50 and 200 ppm produced thinner hyphae, whereas concentrations ≥300 ppm induced irregular thickening.

In *T. harzianum*, the modifications were less pronounced. From 450 ppm onward, an initial reduction in hyphal diameter was observed, followed by a gradual increase as the concentration of CuO NPs increased.

**Figure 6:**
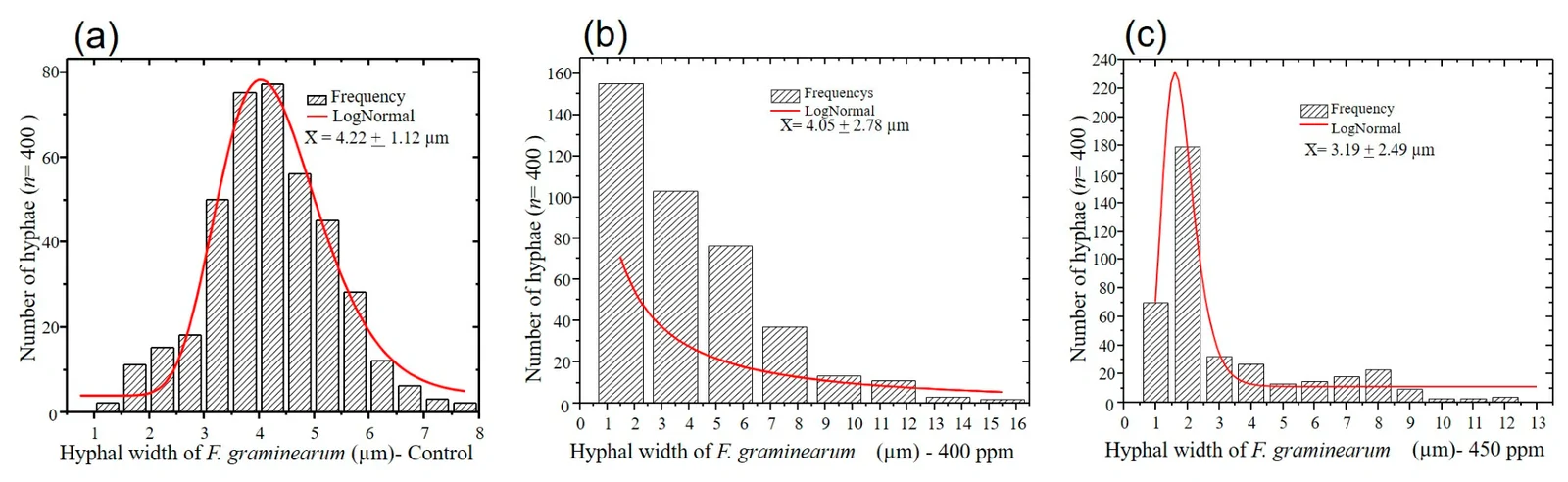
Frequency histograms of hyphal width in *Fusarium graminearum* exposed to CuO NPs (*n* = 400 hyphae per treatment). (**a**) Control; (**b**) 400 ppm; and (**c**) 450 ppm CuO NPs.

The distribution of hyphal width in *F. graminearum* under the control treatment and at CuO NP concentrations of 400 and 450 ppm is presented in Figure 6. Both treatments showed significant differences compared with the control and corresponded to the concentrations at which clear hyphal deformation was observed, prior to complete inhibition at 500 ppm. The control exhibited an approximately normal distribution, with a mean hyphal width of 4.22 ± 1.12 µm (Figure 6a). In contrast, the 400 ppm treatment showed a higher frequency of hyphae with diameters between 0.5 and 2.5 µm and a lower proportion of thick hyphae (14.5–16.5 µm), with a mean width of 4.05 ± 1.12 µm (Figure 6b). At 450 ppm, hyphae smaller than 2.5 µm predominated, with a further reduction in the proportion of thick hyphae (8.5–12.5 µm) and a mean width of 3.19 ± 2.49 µm (Figure 6c).

**Figure 7:**
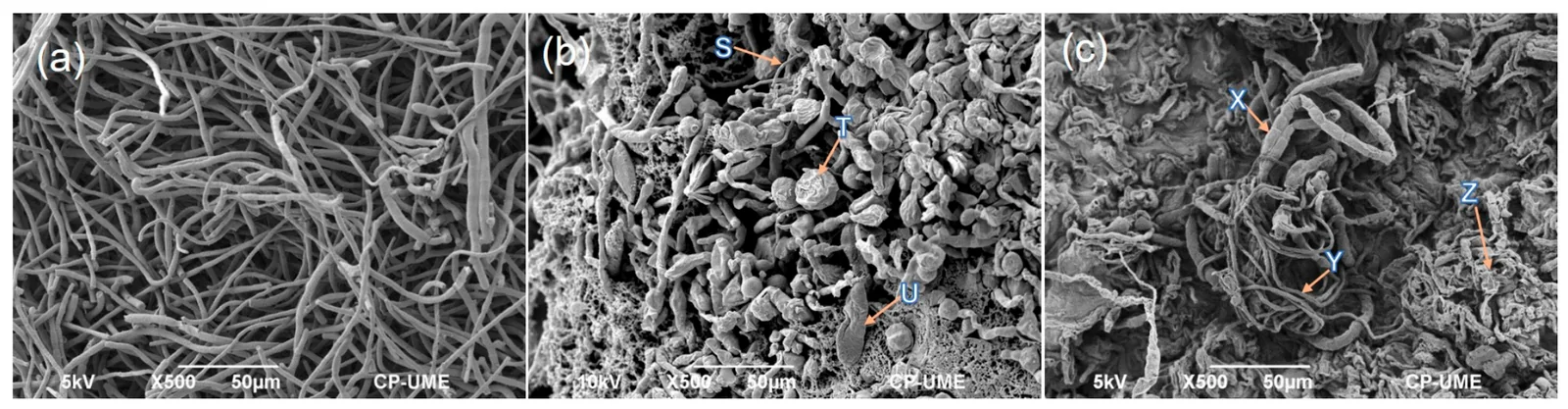
Scanning electron microscopy (SEM; JEOL JSM-6390) micrographs of *Fusarium graminearum* exposed to CuO NPs. (**a**) Control without CuO NPs, showing uniform hyphae with no apparent structural alterations. (**b**) Hyphae exposed to 400 ppm CuO NPs, showing morphological alterations, including thin and deformed hyphae (S), chlamydospore formation (T), irregular hyphal thickening (U), and damage to the mycelial structure. (**c**) Hyphae exposed to 450 ppm CuO NPs, showing more pronounced morphological alterations, including thickened hyphae (X), thin hyphae (Y), and structural collapse (Z). These micrographs illustrate the antifungal effects of CuO NPs on *F. graminearum*.

The SEM micrographs confirmed the changes observed in the morphometric analysis (Figure 7). The control treatment showed uniform hyphae with no apparent structural alterations (Figure 7a). In contrast, samples exposed to 400 and 450 ppm showed hyphal deformation and collapse, surface damage to the cell wall, and chlamydospore formation (Figure 7b,c).

**Figure 8:**
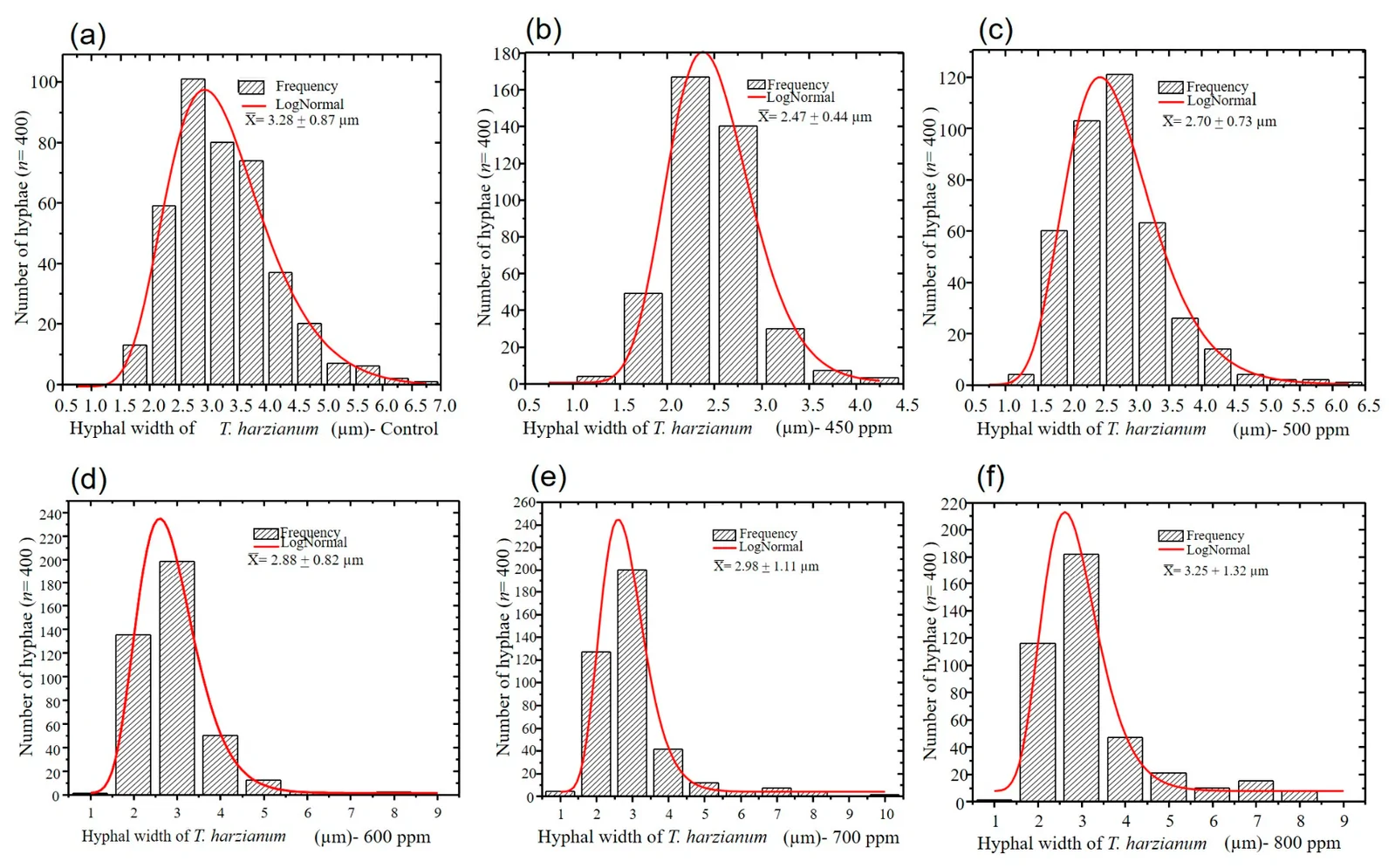
Frequency histograms of hyphal width in *Trichoderma harzianum* exposed to CuO NPs (*n* = 400 hyphae per treatment). (**a**) Control; (**b**) 450 ppm; (**c**) 500 ppm; (**d**) 600 ppm; (**e**) 700 ppm; and (**f**) 800 ppm CuO NPs.

The distribution of hyphal width in *T. harzianum* exposed to 450–800 ppm CuO NPs is shown in Figure 8. All treatments differed significantly from the control in both radial growth and hyphal width. The control exhibited a mean hyphal width of 3.28 ± 0.87 µm (Figure 8a), whereas the treatments with 450, 500, 600, 700, and 800 ppm showed mean values of 2.47 ± 0.44, 2.70 ± 0.73, 2.88 ± 0.82, 2.98 ± 1.11, and 3.25 ± 1.32 µm, respectively (Figure 8b–f). In all treatments, the highest frequency of hyphae was concentrated between 2.0 and 3.5 µm, whereas concentrations of 600–800 ppm showed a lower proportion of hyphae with diameters between 7.5 and 10.5 µm.

**Figure 9:**
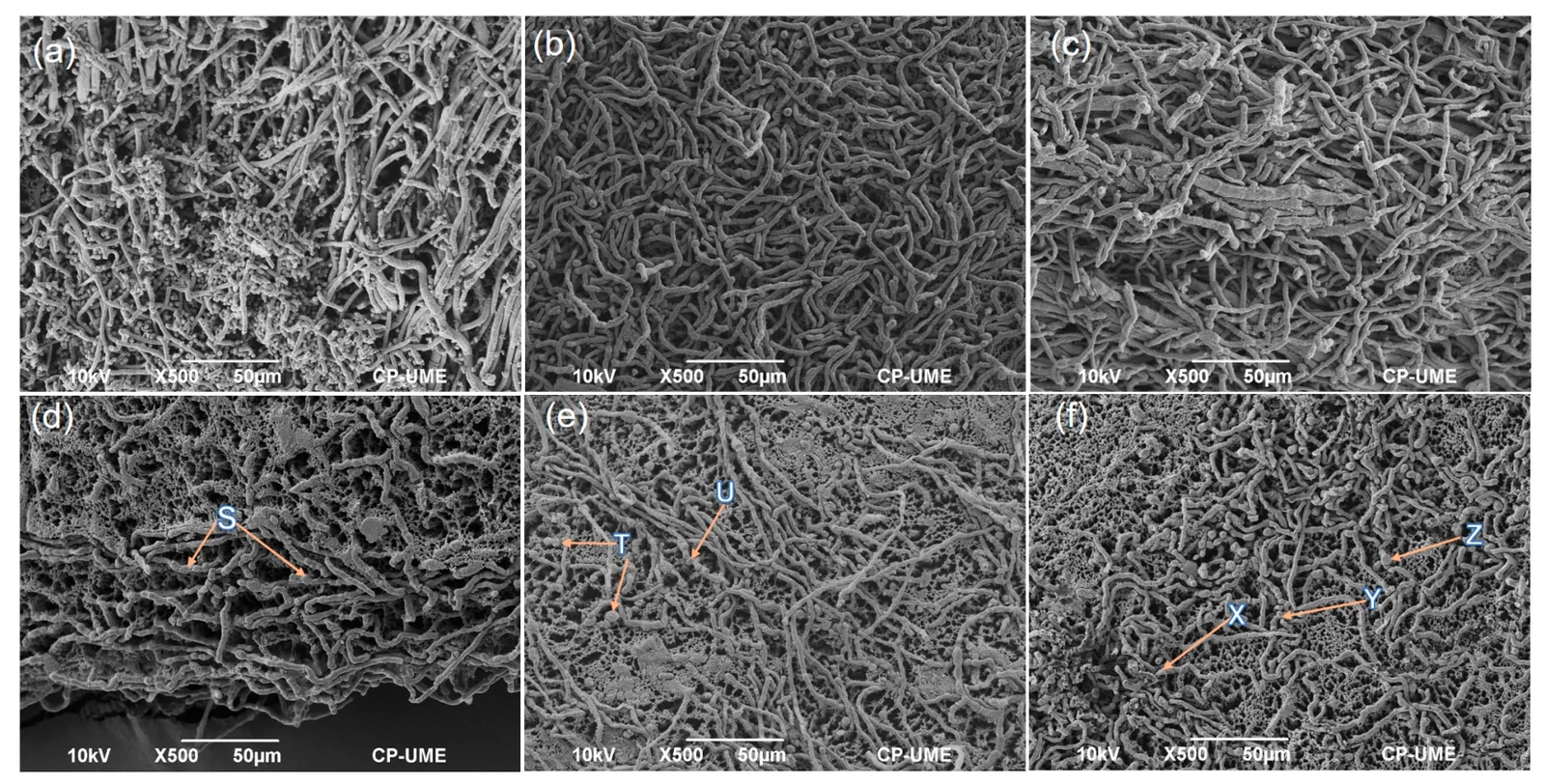
Scanning electron microscopy (SEM; JEOL JSM-6390) micrographs of *Trichoderma harzianum* exposed to CuO NPs. (**a**) Control, showing uniformly tubular hyphae without apparent structural alterations and abundant conidia. (**b**,**c**) Hyphae exposed to 450 and 500 ppm CuO NPs, respectively, showing a reduced presence of conidia. (**d**) Hyphae exposed to 600 ppm CuO NPs, showing reduced mycelial density and the formation of chain-like structures (S). (**e**) Hyphae exposed to 700 ppm CuO NPs, showing reduced mycelial density, chlamydospore formation (T), and chain-like structures (U). (**f**) Hyphae exposed to 800 ppm CuO NPs, showing structural damage (X), chain-like structures (Y), and chlamydospore formation (Z). The micrographs show progressive morphological alterations associated with increasing CuO NP concentrations.

The SEM micrographs revealed morphological differences associated with increasing CuO NP concentrations (Figure 9). The control showed abundant mycelial development and conidiation (Figure 9a). In the treatments with 450 and 500 ppm, adequate hyphal density was maintained, and no evident structural alterations were observed; however, the number of conidia was lower than that in the control (Figure 9b,c). At concentrations of 600, 700, and 800 ppm, lower mycelial density, reduced conidiation, chain-like structures, and chlamydospore formation were observed (Figure 9d–f).

### 3.5. Sporulation Assays of T. harzianum Exposed to CuO NPs

**Figure 10:**
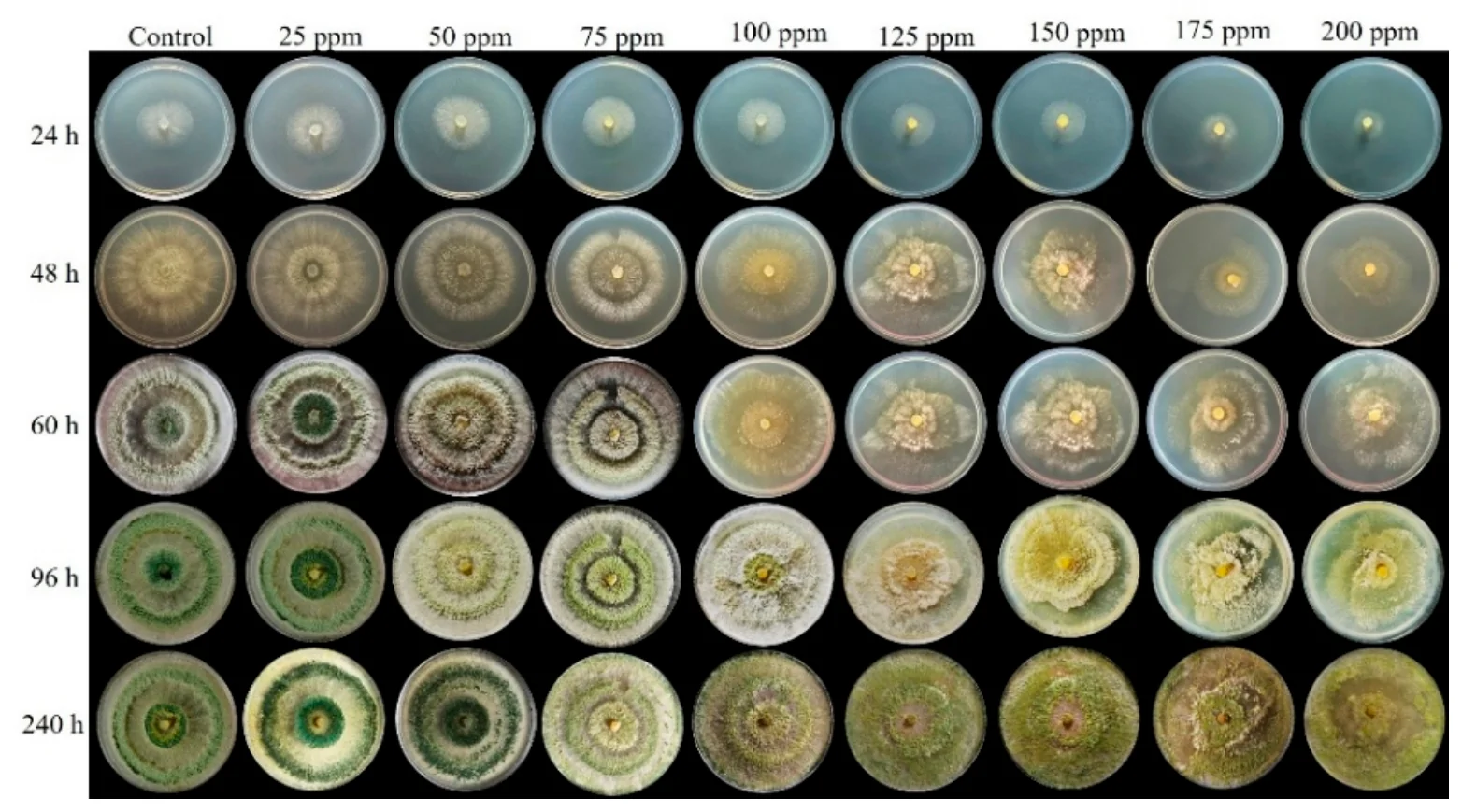
Monitoring of *Trichoderma harzianum* sporulation on Difco™ PDA medium over a 10-day incubation period following exposure to different concentrations of CuO NPs.

The effect of different concentrations of CuO NPs on the sporulation of *T. harzianum* was evaluated during a 240 h incubation period. At 60 h, fungal growth in the control and in the treatments with 25, 50, and 75 ppm completely covered the surface of the culture medium. Furthermore, these treatments visually exhibited higher conidial production compared with the control (Figure 10).

**Figure 11:**
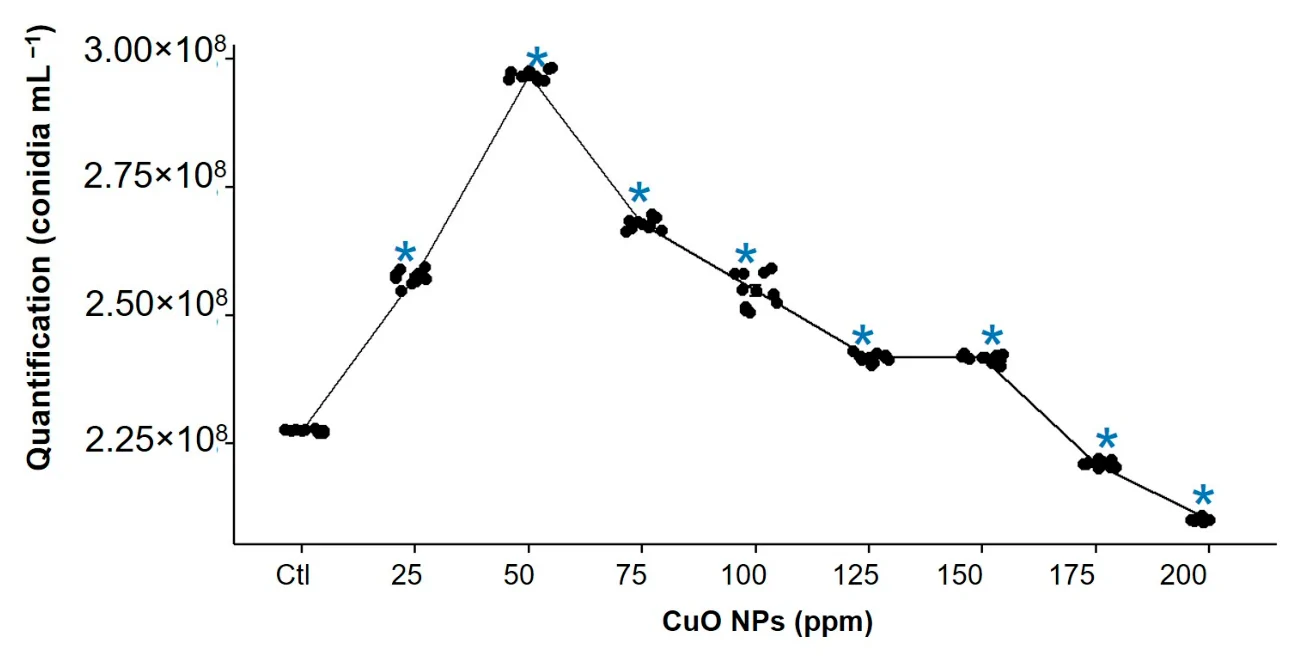
Sporulation of *Trichoderma harzianum* expressed as conidia mL^−1^ following exposure to different concentrations of CuO NPs. * Indicates significant differences from the control according to the Kruskal–Wallis test (*p* < 0.05; *n* = 10).

Conidium quantification confirmed these differences (Figure 11). The control treatment had an average concentration of 2.28 × 10^8^ conidia mL^−1^. Conidial production increased to 2.58 × 10^8^ conidia mL^−1^ at 25 ppm and reached its maximum value at 50 ppm, with 2.97 × 10^8^ conidia mL^−1^, representing a 30.4% increase compared with the control. Starting at 75 ppm, conidium production gradually decreased as the concentration of CuO NPs increased, with values of 2.68 × 10^8^, 2.55 × 10^8^, and 2.42 × 10^8^ conidia mL^−1^ recorded at 75, 100, and 125 ppm, respectively. The lowest production was observed at 200 ppm, with 2.10 × 10^8^ conidia mL^−1^, corresponding to a 7.6% reduction compared with the control.

**Figure 12:**
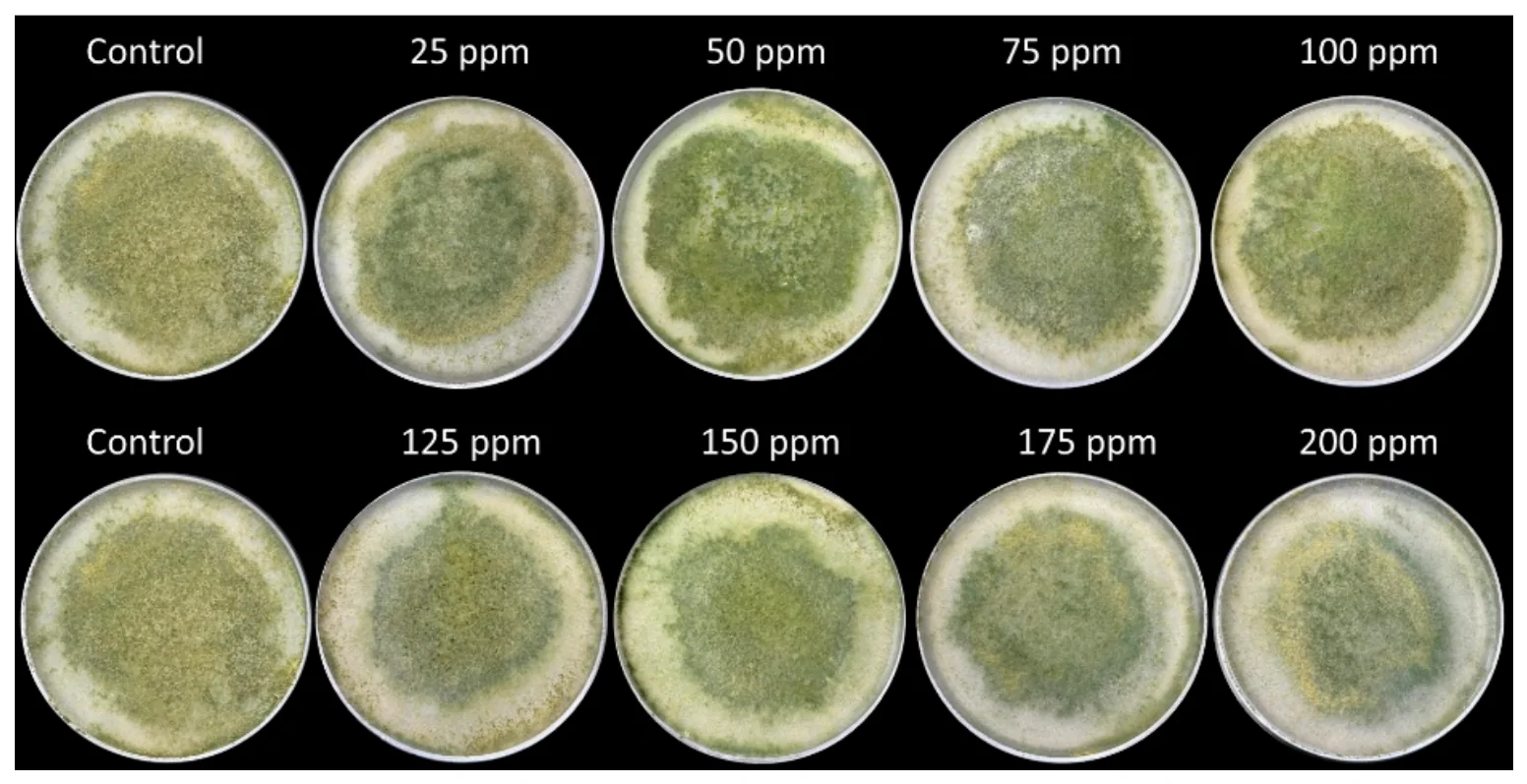
Germination of *Trichoderma harzianum* conidia on Difco™ PDA medium after 72 h of incubation. A suspension containing 1 × 10^6^ conidia mL^−1^, recovered from cultures exposed to 25–200 ppm CuO NPs and from the control treatment, was used (*n* = 3).

The conidia recovered from all treatments germinated normally after 72 h of incubation (Figure 12). No differences were observed in mycelial growth establishment compared with the control, suggesting that the concentrations evaluated did not affect conidial viability.

## 4. Discussion

### 4.1. Synthesis, Structural and Morphological Characterization, and Composition of CuO Nanoparticles Using SEM, TEM, EDS, Raman, and XRD

Chemical precipitation synthesis yielded CuO nanoparticles with an average yield of 94.33%. This method stands out for its simplicity, low cost, and reproducibility characteristics that have been widely reported for the preparation of metal nanoparticles and metal oxides [52,53].

The micrographs obtained by SEM and TEM (Figure 1) showed nanoparticles with a predominantly quasi-spherical morphology, consistent with previous studies that employed similar chemical precipitation methodologies [52,54]. Elemental analysis by EDS (Figure 1b) confirmed the presence of copper and oxygen, corroborating the formation of CuO. The differences observed in the elemental composition compared with other studies [52,54,55] can be attributed to instrumental variations, sample preparation conditions, or substrate contributions during analysis [56].

The average size of 10.9 ± 0.7 nm determined by TEM (Figure 1d) falls within the range reported by [54] for nanoparticles synthesized using this method. Likewise, the crystallite size obtained by XRD (9.6 ± 0.7 nm) showed close agreement with that determined by TEM, supporting the reliability of both techniques for characterizing nanoscale materials [57]. This agreement also suggests that the particles consist primarily of single crystals or a small number of crystalline domains. Furthermore, sizes below 20 nm are typically associated with a higher specific surface area and greater chemical reactivity, characteristics relevant to biological and environmental applications [58,59].

Raman and XRD analyses (Figure 2) confirmed the formation of the monoclinic tenorite phase of CuO, with no evidence of secondary phases, indicating high crystalline purity of the synthesized material [40,44]. Furthermore, refinement using spherical harmonics allowed for the estimation of an ellipsoidal crystallite morphology, serving as a complementary tool for correlating crystallographic information with the morphology observed by electron microscopy.

Several studies have shown that CuO nanoparticles smaller than 20 nm possess a high surface-to-volume ratio, which promotes the release of Cu^2+^ ions and the generation of ROS, both of which are closely related to their antimicrobial activity [44,59,60]. In this context, the physicochemical characteristics obtained in this study provide a basis for interpreting the marked antifungal activity observed against *F. graminearum* and the tolerance exhibited by *T. harzianum*.

### 4.2. Inhibitory Potential of CuO Nanoparticles Against Fusarium graminearum

In our work, we observed an antifungal activity of CuO NPs against *F. graminearum* that is concentration-dependent, beginning at 300 ppm and reaching complete inhibition at 500 ppm, which is consistent with previous studies. Kong et al. reported that CuO NPs size influenced antifungal efficacy and documented effects associated with hyphal growth, conidial viability and germination, oxidative stress, and membrane integrity [37]. In addition, CuO NPs synthesized from a cell-free filtrate of *T. harzianum* exhibited antifungal activity against *F. graminearum* at 0.034 nM [61]. It has also been reported that CuO NPs inhibit the growth of *F. oxysporum* across a wide concentration range (from 25 to 500 ppm) in a dose-dependent manner [62,63]. On the other hand, CuO NPs inhibit the growth of *F. solani* across widely differing concentration; while one study observed this effect in a range of 50 to 100 µg/L [64], another study reported similar effects in a range of 50 to 250 mg/L [65]. Although all these studies show a dose-dependent antifungal effect of CuO NPs, the ranges at which this effect is observed vary across studies; this may be due to nanoparticle size, the synthesis method, the use of stabilizing agents, and the specific interaction between the nanoparticle and the fungal species being analyzed.

The complete inhibition of the radial growth of *F. graminearum* at 500 ppm (Figure 3) is consistent with previous studies reporting susceptibility of the genus *Fusarium* to copper-based nanomaterials [37,61,62,63,64,65,66,67,68]. Although the specific biochemical and molecular pathways were not directly quantified in this study, existing literature suggests plausible mechanisms for this antifungal effect. It is widely hypothesized that this toxicity may be primarily driven by the release of cupric ions (Cu^2+^), which can replace essential ions in enzymes and proteins, impairing their function and causing cellular stress [21]. Furthermore, previous reports indicate that intracellular accumulation of ROS, such as hydroxyl radicals (OH) and superoxide anions (O_2_^−^), likely contributes to the antifungal activity by promoting the peroxidation of polyunsaturated fatty acids, altering membrane potential, and causing the loss of cytoplasmic content [18,20,62].

Several authors have also reported that small CuO NPs may penetrate the nuclear envelope and cause DNA damage, while the released Cu^2+^ ions could compromise the integrity of cell membranes and cellular structures [18,20,62]. In this study, while direct measurements of ion release, ROS generation, or specific membrane disruptions were not performed, the morphological alterations observed by SEM, including hyphal deformation, structural collapse, and superficial damage to the cell wall are consistent with these previously proposed mechanisms. Furthermore, it has been reported that nanoparticle deposition on the mycelial surface may interfere with the synthesis of chitin and β-glucans, weakening the cell wall and compromising fungal viability [18,64,69,70].

### 4.3. Tolerance Mechanisms in Trichoderma harzianum

In contrast to *F. graminearum*, *T. harzianum* exhibited remarkable tolerance to CuO NPs, maintaining active mycelial growth at concentrations as high as 800 ppm (Figure 3). This physiological tolerance highlights the efficient homeostatic and detoxification mechanisms characteristic of the genus *Trichoderma* [71,72]. While molecular and biochemical responses were not quantified in this work, existing literature suggests plausible mechanisms for this tolerance. For instance, it has been reported that filamentous fungi of this genus possess ATP-binding cassette (ABC) transporters and major facilitator superfamily (MFS) transporters that have been shown to actively export excess Cu^2+^ ions from the cytosol [73,74]. Furthermore, intracellular chelation of heavy metals by cysteine-rich metallothioneins and phytochelatins is thought to prevent the accumulation of free ions capable of triggering ROS generation [75,76]. Extracellular barriers, such as the secretion of exopolysaccharides (EPS) and organic acids, may also restrict the initial uptake of nanoparticles into the intracellular environment [77,78]. One or more of these previously proposed tolerance mechanisms may contribute to the ability of *T. harzianum* to grow at higher concentrations of CuO NPs than *F. graminearum*; however, direct experimental evidence is required to elucidate the specific mechanisms underlying this differential tolerance in our model.

The tolerance of *T. harzianum* to CuO NPs observed in this study should also be considered in the context of previous research on copper-based nanomaterials. A previous study shows that metallic copper nanoparticles at 100 mg/L did not significantly inhibit the mycelial growth of *T. harzianum,* whereas the same concentration accelerated and increased its sporulation rate. However, a high concentration of CuO NPs (1000 mg/L) completely inhibited the growth of the fungus [38]. It has also been reported that CuO NPs at concentrations of up to 200 ppm did not affect the growth of *T. asperellum*; however, a delay in conidiation was observed at 100 and 200 ppm. Furthermore, at 300 and 500 ppm of CuO NPs only a slight change in the pattern of conidiation was observed [79]. This response is consistent with the ability of *Trichoderma* to tolerate copper-based materials in both their nano and bulk forms [38,39,79]. An important contribution of this study is that the antifungal activity of the same CuO NPs was evaluated against both *F. graminearum* and *T. harzianum*; this allowed us to compare the sensitivity of a plant pathogen with the tolerance of a beneficial fungus under the same experimental conditions.

Although CuO NPs did not completely inhibit the fungal growth of *T. harzianum*, they induced morphological changes, including a decrease in mycelial density, reduced conidiation, and the formation of chain-like structures and chlamydospores, particularly at concentrations above 600 ppm (Figure 5b, Figure 8 and Figure 9). These morphological alterations suggest that fungal cells may undergo a metabolic trade-off, redirecting energy resources from vegetative growth toward stress mitigation. Although the activity of specific antioxidant enzymes was not measured in this study, it is plausible based on previous reports that such stress responses involve the activation of antioxidant defenses, including enzymes such as superoxide dismutase (SOD), catalases (CAT), and peroxidases (POD) [80,81,82].

Certain experimental constraints should be considered when interpreting these *in vitro* findings. First, distinct visual alterations in the culture medium, including brownish and greenish colorations, occurred across both inoculated treatments and uninoculated controls. Because these changes manifested in the absence of fungal mycelium, they cannot be explained by fungal pigmentation alone. Although potential causes include CuO NPs interaction with medium components or altered dispersion states, these hypotheses were not tested and warrant cautious interpretation. Second, the colloidal stability, sedimentation rate, and aggregation behavior of CuO NPs were not directly measured within the PDA matrix during incubation. Because matrix interactions can influence effective exposure, biological outcomes in this study are reported based on nominal concentrations. Future research should prioritize evaluating nanoparticle stability and dispersion kinetics under biologically relevant culture conditions.

### 4.4. Influence of CuO NP Physicochemical Properties on Fungal Cell Interaction

The antifungal efficacy of metal oxide nanoparticles is widely recognized to be influenced not only by their chemical composition but also by their physicochemical properties, particularly surface morphology and particle size. The specific surface area, which is inversely proportional to particle size, is generally considered to play a critical role in determining the extent of physical contact with the fungal cell wall and the kinetics of Cu^2+^ ion dissolution [83,84]. In our study, the synthesized CuO NPs exhibited a primary size of 10.9 ± 0.7 nm and a quasi-spherical shape. It is plausible that this nanoscale dimension provides a relatively high density of surface atoms and exposed crystal facets, which could serve as reactive anchoring sites on the fungal cell surface [83].

In the context of our comparative study, it is reasonable to hypothesize that the specific surface morphology and reactivity of the prepared CuO NPs may have contributed to stronger adhesion and localized oxidative/ionic stress at the hyphal apices of *F. graminearum*, potentially playing a role in the severe morphological disruption, hyphal swelling, and growth inhibition observed. Conversely, the differential tolerance of *T. harzianum* suggests that its cell wall architecture (e.g., a potentially thicker chitin layer or specific cross-linking) alongside its robust intracellular antioxidant and efflux systems may effectively mitigate the surface-mediated stress imposed by the CuO NPs. Therefore, the observed differential antifungal activity might be interpreted as a consequence of the complex interplay between the specific surface reactivity of the CuO NPs and the intrinsic structural and physiological defenses inherent to each fungal species.

### 4.5. Differentiation of Chlamydospores as a Stress-Induced Survival Strategy

A key adaptive response shared by both fungal species under sublethal stress was the differentiation of the vegetative mycelium into chlamydospores, observed at 400–450 ppm for *F. graminearum* (Figure 7b,c) and at higher doses (600–800 ppm) for *T. harzianum* (Figure 9d,f). Chlamydospores are thick-walled, lipid-rich resistance structures whose synthesis is governed by evolutionarily conserved mitogen-activated protein kinase (MAPK)-signaling pathways [85,86]. The accelerated formation of these propagules confirms that CuO NPs create a hostile microenvironment, forcing the fungi to shift from vegetative growth to a latent survival state to protect their genetic material from oxidative degradation [20,81].

### 4.6. CuO Nanoparticles Stimulate the Conidiogenesis of Trichoderma harzianum at Low Doses

A concentration-dependent biphasic response was observed in the sporulation of *T. harzianum* following exposure to CuO NPs. Concentrations between 25 and 150 ppm stimulated conidial production relative to the untreated control, whereas higher concentrations progressively reduced reproductive output (Figure 10 and Figure 11). This behavior is consistent with a hormetic-type response, characterized by stimulation at lower exposure levels and inhibition at higher levels [87]; however, it should be noted that formal mathematical dose–response modeling was not performed in this work to quantify the hormesis zone. Previous work by Banik and Pérez-de-Luque [38] also reported a temporary enhancement of *T. harzianum* sporulation following exposure to a low concentration of metallic copper nanoparticles. However, another study reported that exposure to 100 and 200 ppm of CuO NPs delays the conidia formation in *T. asperellum* [39], suggesting that this biphasic response may vary depending on the *Trichoderma* species analyzed. Nevertheless, the mechanisms underlying this response, including potential contributions from copper availability and oxidative signaling, remain hypotheses that require direct experimental verification.

At low concentrations, copper acts as an essential micronutrient. It serves as a necessary cofactor for critical fungal metalloenzymes, such as laccases and copper-zinc superoxide dismutases (Cu/Zn-SOD), which are directly involved in cell differentiation, cell wall pigmentation, and conidial maturation [88,89,90]. At the same time, several studies have shown that moderate levels of reactive oxygen species (ROS) act as intracellular signals that promote differentiation and reproductive development in *Trichoderma* [91]. During the response to environmental stimuli such as mechanical damage, mild oxidative stress, or nutrient limitation, NADPH oxidases generate ROS that activate MAPK-dependent signaling pathways involved in conidiogenesis [92]. This phenomenon corresponds to a eustress-type response (moderate oxidative stress), in which controlled concentrations of ROS function as second messengers that regulate genes associated with asexual development, promoting the formation and maturation of conidia without compromising cell germination [93,94]. Thus, the observed biphasic effect of CuO NPs on *T. harzianum* could result from either of these two mechanisms or even from their combined action.

### 4.7. Spore Germination and Agricultural Implications for Integrated Disease Management

After 72 h of incubation, the methodology employed did not reveal any differences in the germination and vegetative growth of conidia previously exposed to CuO NPs (Figure 12). These results suggest that the morphological alterations observed in *T. harzianum* hyphae during exposure to CuO NPs are not necessarily reflected in the germination and development of the subsequent generation [95,96]. Several studies have shown that *Trichoderma* species possess a high capacity to adapt to metal nanoparticles, which has been attributed to efficient metal homeostasis systems, antioxidant mechanisms, and the regulation of oxidative stress, allowing the restoration of normal growth once the stressor is removed [82,95,97]. Furthermore, *T. harzianum* has been reported to maintain its ability to grow and sporulate following exposure to metal nanoparticles and metal oxides, demonstrating remarkable phenotypic plasticity and tolerance to these materials [95,96].

From an agronomic perspective, the differential *in vitro* response of these two fungi to CuO NPs highlights a potential, yet unproven, avenue for sustainable integrated disease management strategies [64,67,97]. However, it is crucial to explicitly acknowledge the limitations of extrapolating *in vitro* findings to complex agricultural environments. In natural soil, factors such as organic matter binding, clay interactions, and microbial adsorption drastically alter the dissolution, bioavailability, and toxicity dynamics of copper-based nanoparticles [98,99]. Furthermore, while *T. harzianum* demonstrated survival up to 800 ppm in this study, significant reductions in mycelial density and conidiation were observed at concentrations ≥ 600 ppm. Therefore, while the differential sensitivity observed *in vitro* provides a rationale for combining these agents, validating their synergistic potential in agricultural conditions will require further research addressing their direct antagonistic interactions in the presence of CuO NPs.

## 5. Conclusions

Copper oxide nanoparticles (CuO NPs) synthesized by chemical precipitation exhibited marked antifungal activity against *Fusarium graminearum*, whereas *Trichoderma harzianum* showed moderate tolerance to the concentrations evaluated. While these *in vitro* results highlight the promising potential of CuO NPs as a complementary tool for integrated disease management, they must be interpreted with caution regarding field applications. The complexity of agricultural environments will inevitably alter nanoparticle bioavailability and fungal interactions. Therefore, further field studies are needed to validate their efficacy and selectivity, as well as to assess their potential effects on non-target organisms and the environment.

## Data Availability

The original contributions presented in this study are included in the article. For any additional inquiries, please contact the corresponding authors.

## Supplementary Materials

The following supporting information can be downloaded at https://www.mdpi.com/article/10.3390/nano16211396/s1. Figure S1. SEM micrographs of CuO nanoparticles synthesized by chemical precipitation.
