# Supplementary material for: Synthesis and Characterization of CuO Nanoparticles with Differential Antifungal Activity Against Fusarium graminearum and Trichoderma harzianum
Source: Nanomaterials (Basel). 2026 Oct 31;16(21):1396. doi: 10.3390/nano16211396 (PMC13765052; doi:10.3390/nano16211396)
Supplement: Supplementary file 1 [file nanomaterials-16-01396-s001.zip › nanomaterials-4619375-supplementary.pdf]

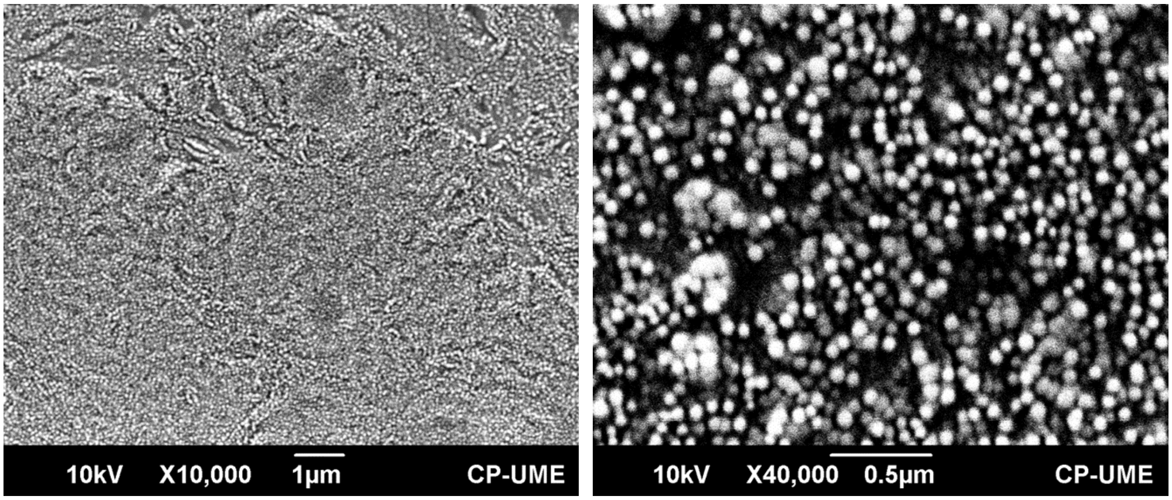

(**a**) (**b**)

Figure S1. SEM micrographs of CuO nanoparticles synthesized by chemical precipitation, acquired using a JEOL JSM-6390 microscope at **a)** 10,000× and **b)** 40,000× magnification, showing their quasi-spherical morphology.
